# Baroreceptor activation attenuates attentional effects on pain-evoked potentials

**DOI:** 10.1016/j.pain.2010.09.028

**Published:** 2010-12

**Authors:** Marcus A. Gray, Ludovico Minati, Giulia Paoletti, Hugo D. Critchley

**Affiliations:** a Clinical Imaging Sciences Centre (CISC), Brighton and Sussex Medical School (BSMS), University of Sussex, Brighton, East Sussex, BN1 9RR, UK; b Experimental Neuropsychology Research Unit, School of Psychology and Psychiatry, Monash University 3800, Australia; c Scientific Department, Fondazione IRCCS Istituto Neurologico “Carlo Besta”, Milano, Italy; d Department of Physiology, University of Siena, Italy; e Sussex Partnership Foundation (NHS) Trust, Sussex, UK

**Keywords:** Baroreceptor, Blood pressure, Cardiac timing, Pain, Evoked potentials, Attention, Homeostasis

## Abstract

Focused attention typically enhances neural nociceptive responses, reflected electroencephalographically as increased amplitude of pain-evoked event-related potentials (ERPs). Additionally, pain-evoked ERPs are attenuated by hypertension and baroreceptor activity, through as yet unclear mechanisms. There is indirect evidence that these two effects may interact, suggesting that baroreceptor-related modulation of nociception is more than a low-level gating phenomenon. To address this hypothesis, we explored in a group of healthy participants the combined effects of cue-induced expectancy and baroreceptor activity on the amplitude of pain-evoked ERPs. Brief nociceptive skin stimuli were delivered during a simple visual task; half were preceded by a visual forewarning cue, and half were unpredictable. Nociceptive stimuli were timed to coincide either with systole (maximum activation of cardiac baroreceptors) or with diastole (minimum baroreceptor activation). We observed a strong interaction between expectancy and cardiac timing for the amplitude of the P2 ERP component; no effects were observed for the N2 component. Cued stimuli were associated with larger P2 amplitude, but this effect was abolished for stimuli presented during baroreceptor activation. No cardiac timing effect was observed for un-cued stimuli. Taken together, these findings suggest a close integration of cognitive–affective aspects of expectancy and baroreceptor influences on pain, and as such may cast further light on mechanisms underlying mental and physiological contributions to clinical pain.

## 1 Introduction

Pain expectation mobilises attentional resources toward relevant external and internal stimuli facilitating adaptive behavioral and physiological responses. Attention modulates both subjective experience [5] and neural correlates [42] of pain, directing attention toward pain, relative to non direction or distraction, and increases pain-evoked ERP amplitudes [8,34,50,75,76]. Similarly, both hypnotic suggestion and expectations about pain modulate electroencephalographic [17] and functional imaging indices of nociceptive processing [41,68]. Thus the neural responses that track subjective pain [14,18,43] do not simply reproduce energy delivered by nociceptive stimuli, but are shaped by cognitive and affective processes [41,55].

Nociceptive processing is also influenced by visceral state. Even within the short timeframe of the cardiac cycle, nociceptive stimuli can be attenuated by discharge of cardiac and arterial baroreceptors, activated naturally at systole by phasic ejection of blood from the heart [3,13,35]. Experimentally, increasing baroreceptor discharge through artificial stimulation (phase related external suction, PRES, over the neck in the carotid region [23,27,59]) typically reduces subjective pain ratings [4,11,22,45,54], without necessarily affecting pain detection thresholds [22,45]. Baroreceptor activity similarly modulates neural signatures of pain processing: PRES, coupled to baroreceptor activation occurring naturally during cardiac systole, engenders a negative shift in pain-evoked ERPs [60], and timing nociceptive stimuli in relation to natural systolic baroreceptor discharge influences the amplitude of the N2 and P2 components of pain-evoked ERPs [4,11,26,54]. Baroreceptor discharge also influences skeletomotor [59] and autonomic reflexes to nociceptive stimulation, inhibiting activity in sympathetic nerves supplying skeletal muscles (muscle sympathetic nerve activity; MSNA) [19], an effect associated with attenuated blood pressure responses to pain [20,21,39,72]. Thus nociceptive processing can be modulated by baroreceptor activation, as evidenced by alterations in subjective reports, pain-evoked potential amplitudes and autonomic reactions.

Influential theories suggest a central role of visceral afferent information in emotion and motivation [15,16,58], yet it is unclear if baroreceptor influences extend beyond cardiovascular homoeostasis or low-level sensory gating. Nociceptive processing provides a unique window to explore how baroreceptor activity might interact with cognitive and motivational functions. Of direct relevance is the observation by Donadio and co-workers that *infrequent* nociceptive stimuli presented during baroreceptor discharge have the greatest selective impact on autonomic reactions (enhancing MSNA inhibition without altering sympathetic skin responses). Moreover, MSNA inhibition rapidly habituates if stimuli are repeated over five consecutive cardiac cycles [20]. One interpretation is that the initial nociceptive stimulus carries attentional salience, amplifying baroreceptor inhibition of MSNA, but subsequent stimuli lose salience and have diminished baroreceptor-related effects. Alternatively, the effect may emerge from refractory characteristics of homoeostatic neurons that specifically fatigue MSNA inhibition across consecutive cardiac cycles.

The current study, extending earlier neuroimaging work [39], was motivated to examine the interaction between attentional salience and phasic visceral effects in nociception. We modulated expectancy by embedding nociceptive stimuli within a visual task, to dissect attentional and baroreceptor influences. We hypothesized that if expectancy and attention, rather than physiological habituation, modulate the baroreceptor gating of pain responses [20], then the baroreceptor influence on the pain-evoked ERPs would be different between expected and unexpected pain, suggesting that attentional effects on central nociception are mechanistically dependent on visceral state.

## 2 Methods

### 2.1 Participants and recording procedure

Eleven adults (age 28 ± 9.8 years) participated in the experiment after providing written informed consent. To avoid recognized gender differences in nociceptive processing [7,31] we restricted our sample to female participants. All participants were medication free at the time of testing and reported no history of psychiatric or neurologic disorders. The study was approved by the Brighton and Sussex Medical School (BSMS) research governance and ethics committee. All recordings were performed in a psychophysiology laboratory, with the participant comfortably seated in a dimly-lit, quiet room while the experimenter remained nearby.

### 2.2 Physiological and EEG recordings

Beat-to-beat blood pressure was recorded through a finger cuff applied on the left hand using the volume-clamp method of Peñáz as implemented by the Finometer device (Finapres Medical Systems BV, Arnhem, The Netherlands). Three lead ECG recordings were made with Ag–AgCl electrodes positioned according to Einthoven’s triangle, standard lead I configuration, using an isolated pre-amplifier (model 1902, CED Ltd., Cambridge, UK). The electrocardiogram (ECG) and blood pressure signals were digitized through a ‘power1401’ data acquisition device (CED) and recorded on a PC running the Spike2 version 7 software.

The electroencephalogram (EEG) was recorded through 19 electrodes positioned according to the 10/20 system and held in place by a lycra cap (Electro-Cap Inc., Eaton OH, USA), using a Mindset MS-24 EEG system (Nolan Computer Systems, Inc., Fort Morgan CO, USA) electrically isolated from all other equipment. After band-pass filtering in the 0.1–30 Hz range, signals were sampled at 512 Hz. Recordings were performed relative to a linked-ears reference, and individual electrode impedances were kept below 5 kΩ.

### 2.3 Experimental task

**Fig. 1:**
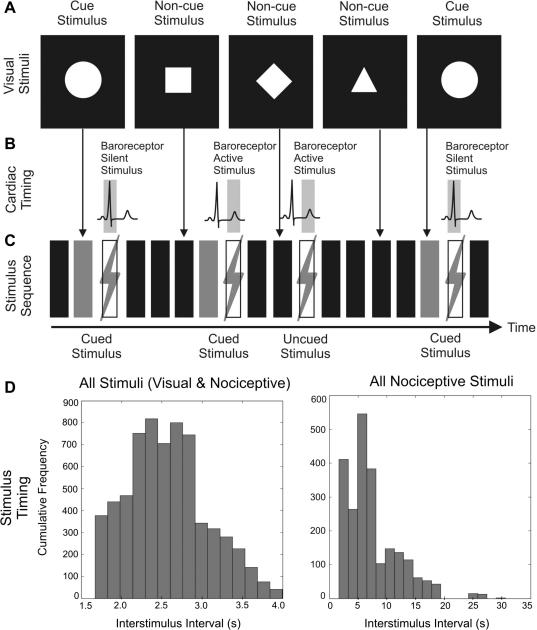
Task design and stimulus frequency. (A) Participants observed four visual stimuli, one of which consistently cued subsequent nociceptive stimuli; in this illustration, the circle acts as the pain cue. (B) Cued and non-cued nociceptive stimuli were timed to coincide with baroreceptor active (300 ms after the ECG R-wave) or baroreceptor silent periods (during the ECG R-wave). (C) Cardiac-paced nociceptive stimuli were either explicitly cued (cues represented here as gray rectangles) or presented without a warning cue. (D) Histograms of the distribution of inter-stimulus intervals. Left: bin centers: 1.74–3.92 s, bin width 1551 ms. Right: bin centers: 2.50–29.82 s, bin width 1608 ms).

Participants performed a passive viewing task, in which four white visual shapes (square, circle, rhombus or triangle) were displayed on a black background for 300 ms by means of a CRT screen positioned at 1 m distance. They were initially shown each stimulus shape and the experimenter indicated which one, randomly chosen for each session, would be the ‘target’ and would therefore always be followed by a nociceptive electrical skin stimulus (see Fig. 1A–C). Targets accounted for 25% of visual stimuli. Nociceptive stimuli were timed to either coincide with the ECG R-wave or to be delivered 300 ms after it. The R-wave corresponds to the end of cardiac diastole, and therefore to relative baroreceptor inactivity. On the contrary, 300 ms after the R-wave corresponds approximately to the systole, when baroreceptor firing is maximal. In other words, in order to perform this study we did not measure the latency of baroreceptor activation with respect to the R-wave, but we assumed these timings a priori, on the basis of the convergent findings reported in existing literature [25–28,39,53].Throughout this paper, stimuli presented during the ECG R-wave are termed “baroreceptor silent stimuli” whereas stimuli presented 300 ms following the ECG R-wave are termed “baroreceptor active stimuli”. The timing of the electrical stimuli was controlled by a real-time script running on the CED-power1401 unit, identifying the QRS complex with sub-millisecond temporal accuracy. Participants were informed that, in addition to the nociceptive stimuli cued by the visual target, un-cued stimuli would also be presented at unpredictable points throughout the task.

A complicating factor is the possibility of expectancy-induced cardiovascular responses, since increased blood pressure during pain anticipation may attenuate pain processing [24,48,62]. Consequently, the average time between the cue and the electrical stimulus was kept brief (3.1 ± 0.3 s), corresponding to a jitter of about 10% which included the variable delay due to cardiac synchronization. Additionally, jittering the cue-to-nociceptive-stimuli interval ensured that the effect of cue-induced expectancy on the ERPs could not be confounded by synchronization of EEG rhythms potentially induced by the visual stimuli. Considering all stimuli (visual and electrical) together, the average inter-stimulus time was 2.6 ± 0.5 s; separately, visual stimuli occurred every 3.9 ± 1.7 s, and nociceptive electrical stimuli every 7.4 ± 4.5 s. We specifically ensured nociceptive stimuli were never delivered during consecutive cardiac cycles to minimise the possible refractory attenuation of baroreceptor influences. The corresponding distributions are shown in Fig. 1D.

Participants completed the task in four blocks of about 380 s each, with a pause of approximately 140 s between blocks. Painfulness ratings for the electrical-skin stimuli were verbally collected after each block on a 1 (barely identifiable as pain) to 10 (imaginary worst possible pain) scale, and averaged across blocks. The overall task duration was approximately 30 min. In total, 200 nociceptive electrical-skin stimuli were delivered, evenly balanced between the baroreceptors silent and active conditions, and between the cued and un-cued conditions.

### 2.4 Electrical-skin stimuli

Two standard EEG electrodes (Ag–AgCl, 5 mm radius circular cup filled with Ten20 conductive paste) separated by approximately 1 cm were attached to the right ventral wrist. Electrical-skin stimuli were delivered by means of a constant-current stimulator (DS7A, Digitimer Ltd., Glenwyn Garden City, UK), and consisted of a single square-wave pulse with 2 ms width and maximum voltage 400 V. Before starting the experimental task, stimulus intensity was determined on an individual by individual basis. Beginning at 5 mA which no participants rated as painful, intensity was incrementally increased in 2 mA steps until participants reported perceiving the stimulus as ‘painful’ for three presentations in row. Stimuli were then maintained at these levels during the following experimental task. Across participants, the stimulus intensity was 17 ± 8 mA.

### 2.5 Data analysis

**Fig. 2:**
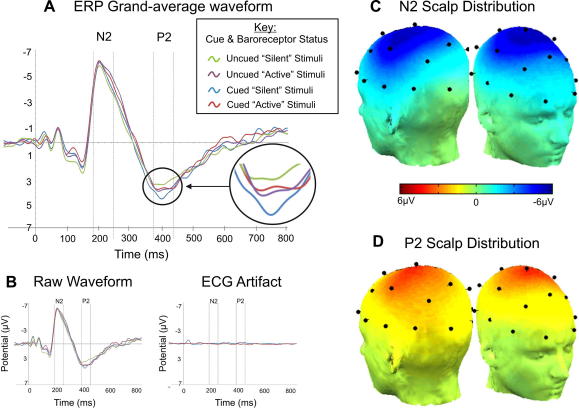
Grand-average waveforms of the event-related potentials evoked by nociceptive stimulation and corresponding scalp distributions. (A) Grand average (across all participants and the C3, Cz and C4 sites) after removal of the ECG artefact. (B) Grand average prior to artefact removal (left) and corresponding ECG artefact (right). (C) Scalp distribution of the N2 component. (D) Scalp distribution of the P2 component.

All EEG channels were re-referenced to the mean of all electrodes, and then epoched in the −100 ms to 800 ms peristimulus range. Baseline removal was performed using the 100 ms pre-stimulus level. On the basis of the grand-average traces of all participants and electrodes, the measurement windows were set to 200 ± 40 and 400 ± 40 ms for the N2 and P2 components, respectively. Investigating evoked-potentials within a cardiac-paced design raises the potential for confounding effects due to contamination of the EEG signal by the ballistocardiogram [1], a possibility which no study within this field has to date explicitly considered. Consequently, we specifically measured and removed the ECG-related artefact from our ERP traces. The artefact was determined by averaging each EEG channel over cardiac cycles during which no stimuli were delivered. As in the EPR analysis, baseline removal subtracted consisted of subtracting the mean 100 ms pre-stimulus activity, producing a measure of ECG induced artefacts in each EEG channel, separately for baroreceptor silent and baroreceptor active centered epochs (See Figure 2c). Prior to statistical analysis using the N2 and P2 time-windows, the resulting averaged artefact was subtracted from the evoked-potentials recorded for the electrical stimuli.

The arterial pressure signal was low-pass filtered at 10 Hz and pre-processed with a peak-picking algorithm which yielded mean arterial pressure (MAP), systolic and diastolic values for each beat. The ECG signal was low-pass filtered at 50 Hz and pre-processed with an algorithm identifying each R-wave and yielding a heart-rate value for each beat. For all physiological measures, polynomial detrending was performed over the entire session using a third-degree polynomial.

In order to evaluate the physiological effect of nociceptive electrical-skin stimuli, changes in MAP and heart rate were calculated for the three post-stimulus beats with respect to the average of the three beats preceding each stimulus. We also measured physiological changes immediately preceding the nociceptive stimulus elicited by the visual warning cue, by considering the difference between the two beats preceding the electrical stimulus and the −3.5 to −4.5 s pre-stimulus period; this temporal window always preceded presentation of the visual warning cue. To determine whether this difference was statistically significant, we compared it with that observed for un-cued pain stimuli by means of two-tailed paired *t*-tests.

Statistical analysis of the peripheral physiology and ERP data was performed by ANOVAs, using within-subject factors for cardiac timing (baroreceptors silent or active) and expectancy (cued or un-cued pain). For the ERP analysis, data from the C3, Cz, and C4 sites, at which the N2/P2 component amplitude was the largest (see Section 3), were pooled together introducing an additional factor for site. Further, blood pressure and heart rate measurements as well as painfulness ratings were entered in the analyses as between-subject covariates. Where appropriate, results were subject to Greenhouse–Geisser correction for violation of the sphericity assumption. Further, Bonferroni’s correction was applied to account for multiple comparisons.

## 3 Results

### 3.1 Peripheral physiology and pain ratings

Overall, participants rated the painfulness of the stimuli at 4.1 ± 1.0 (scale 1–10). Averaging over the whole experimental session, the heart rate (HR) was 73 ± 12 bpm, and the systolic, diastolic and mean arterial (MAP) blood pressures were respectively, 127 ± 26, 73 ± 19, 94 ± 24 mm Hg. There was no correlation between painfulness ratings and baseline MAP values (*p* = 0.8). The results of the beat-to-beat analyses of blood pressure and heart rate changes following nociceptive stimulation are given in Table 1. After correcting for multiple comparisons (*α* = 0.004), there were no significant main effects of expectancy or timing, and no interactions. As regards to the physiological correlates of expectancy preceding pain delivery, there were no effects on MAP (0.09 ± 0.26 Δmm Hg vs. −0.09 ± 0.24 Δmm Hg, *t*(11) = 2.1, *p* = 0.06) and HR (0.10 ± 0.68 Δbpm vs. 0.13 ± 0.53 Δbpm, *t*(11) = −0.1, *p* = 0.9).

### 3.2 Effect of physiological changes and perceived painfulness on ERP components

Despite the absence of statistically significant effects of the warning cue on MAP and HR (see above), we examined whether the physiological state immediately preceding nociceptive stimulation, indexed by MAP and HR, modulated the effects of expectancy and cardiac timing on the N2 and P2 component amplitudes.

Considering the N2 component, the amplitude of the pre-stimulus MAP change, entered in the analysis as between-subjects covariate, did not interact with expectancy (*p* = 0.9) or cardiac timing (*p* = 0.9). Likewise, we observed no interaction of HR change with expectancy (*p* = 0.7) or with cardiac timing (*p* = 0.7). Similarly, when considering the P2 component, there was no significant interaction of MAP change with expectancy (*p* = 1) or cardiac timing (*p* = 1). Again, we also observed no interaction of HR change with expectancy (*p* = 0.7) or with cardiac timing (*p* = 0.7). These covariates were therefore removed from subsequent analyses.

In addition to physiological changes, we also examined associations between painfulness ratings and the N2 and P2 amplitudes. For the N2 component, pain ratings did not interact with expectancy (*p* = 0.2) or cardiac timing (*p* = 0.3). Likewise for the P2, painfulness ratings did not interact with expectancy (*p* = 0.9) or cardiac timing (*p* = 0.4). This covariate was therefore removed from subsequent ERP analyses.

### 3.3 Factorial analysis of ERP components

The amplitude of the ECG artefact was minimal compared with that of the evoked responses and the observed artefact decayed within 100 ms after the R-wave, therefore preceding the temporal windows considered in this study (Fig. 2A–B). Statistical analysis confirmed that there was no significant artefact-related bias neither in the N2 window (*p* = 0.5) nor in the P2 window (*p* = 0.1). As shown in Fig. 2C–D, the N2 and P2 components had a central distribution over the scalp surface, with maximum amplitude at the C3, Cz and C4 sites which were considered for all ERP measurements in this study.

**Fig. 3:**
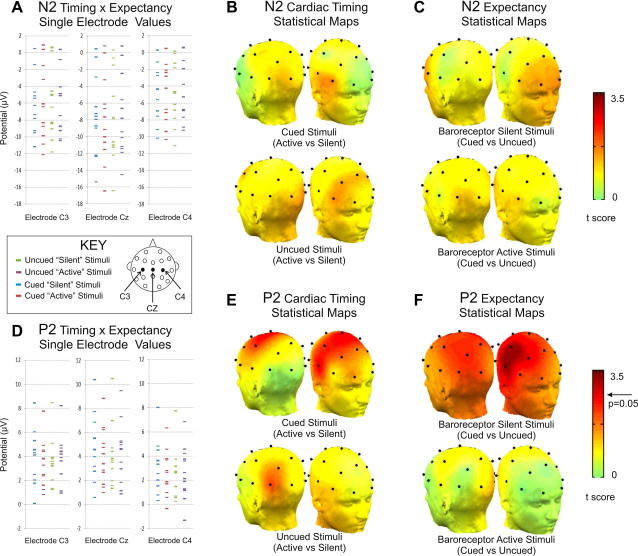
Scatter plots and scalp distributions of statistical significance. For the N2 component, no effects of cardiac timing are observed either on the scatterplot (A) or on the scalp maps of cardiac timing (B) or expectancy effects (C). By contrast, P2 amplitude reveals a significant timing by expectancy interaction (D). Post hoc comparisons exploring cardiac timing (E) reveal increased P2 amplitude when baroreceptors were silent only when stimuli are presented after a cue. There is no cardiac timing effect for un-cued stimuli. The complementary post hoc tests exploring expectancy (F) demonstrate increased P2 amplitude for cued stimuli only when these are presented while the baroreceptors are silent. There is no expectancy effect for stimuli presented during baroreceptor activation.

As regards to the effects of the experimental conditions, for the N2 component there were no main effects of expectancy (*p* = 0.8) and cardiac timing (*p* = 0.7) and no interaction (*p* = 0.4). The corresponding potentials are given in Table 2, and scatter plots and significance maps are shown in Fig. 3A–C.

By contrast, as represented in Fig. 3D, for the P2 component a significant expectancy by cardiac timing interaction was observed (*F*(1,11) = 12.1, *p* = 0.005, $\eta_{p}^{2}=0.52$), without main effects of expectancy (*p* = 0.1) or cardiac timing (*p* = 0.4). We performed post hoc ANOVAs exploring the effect of expectancy on the P2 amplitude separately for stimuli delivered while the baroreceptors were silent, and while baroreceptors were active When considering only baroreceptor silent stimuli, we observed a significant effect of expectancy (*F*(1,11) = 13.2, *p* = 0.004, $\eta_{p}^{2}=0.55$), (see Fig. 3F) with larger amplitude for cued stimuli; there was no significant interaction with side (*p* = 0.8). The corresponding ANOVA for stimuli presented while the baroreceptors were active did not reveal an effect of expectancy (*p* = 0.3), and again no significant lateralization (*p* = 0.4) (Fig. 3F).

Additional post hoc ANOVAs were performed to explore the effect of cardiac timing separately for cued and un-cued stimuli. These revealed that there was a cardiac timing effect for cued (*F*(1,11) = 7.2, *p* = 0.02, $\eta_{p}^{2}=0.40$) but not for un-cued stimuli (*p* = 0.4). The observed effect consisted of an attenuation of the P2 amplitude following cued stimuli presented while the baroreceptors were active; the effect was most strongly significant over the C3, Cz, and C4 sites and was not significantly lateralized (*p* = 0.3, Fig. 3E).

For consistency with previous work, we also examined the N2-P2 difference, for which there were no main effects of expectancy (*p* = 0.3) or cardiac timing (*p* = 0.8) and no interaction (*p* = 0.3).

## 4 Discussion

Our principal finding is an expectancy by cardiac timing interaction on the amplitude of the pain-evoked P2 component. This can be interpreted from two complementary perspectives, through post hoc tests performed either separately for cued and un-cued stimuli, or separately for stimuli presented with active or silent baroreceptors. We found that cue-associated expectation of pain increased the P2 amplitude for stimuli presented without the simultaneous influence of baroreceptor discharge; this effect was abolished when stimuli were presented coincident with baroreceptor discharge. Further, baroreceptor discharge attenuated the P2 amplitude in a statistically significant manner only for cued stimuli; when nociceptive stimuli were unexpected (i.e., participants were attending to the visual stimuli), a trend in the opposite direction was observed, but this was clearly not significant (*p* = 0.4). These results raise intriguing possibilities concerning the mechanisms by which expectancy and phasic visceral signals interact in their influence on pain-evoked potentials. Neither the visual cues indicating imminent nociceptive stimulation nor the stimuli themselves were embodied in significant changes of blood pressure or heart rate, and therefore did not confound the central expectancy-related influences on nociceptive processing. Reassuringly, we found no significant effect of ECG contamination on the ERP traces, enabling us to rule out a potential direct confounding effect related to the ballistocardiogram.

We manipulated pain expectancy by embedding nociceptive stimuli within a visual task including an explicit cue predicting nociceptive stimulation. Cue presentation alters attentional focus, shifting it from the visual stimuli towards imminent painful stimulation. Previous research demonstrates that directed attention increases experienced pain, whereas distraction reduces it [5,63,64], an effect which is also manifest in increased amplitudes of pain-evoked potentials [49–51,55]. Our results are partially in line with these studies. Without the simultaneous influence of baroreceptor activation, cue-induced expectation of pain was associated with increased P2 amplitude, in agreement with the interpretation based on attentional focus. Nevertheless, this effect was absent for stimuli presented during baroreceptor activation. We hypothesize that this is because baroreceptor firing disrupts the attentional modulation; none of the previous studies on attentional modulation explored this effect, as stimuli were not timed to the cardiac cycle. A few limitations should be mentioned: firstly restricting our sample to females restricts the generalizability of findings, and future research could control for effects of menstrual phase on nociception. Secondly, while the time-course of our evoked potentials is consistent with a-delta pain-evoked potentials, electrical-skin stimuli delivered to the ventral wrist may also activate a-beta somato-sensory fibers. More selective a-delta stimulation by laser stimulation of the dorsal hand would reduce the potential for interference by somatosensory evoked potentials. Thirdly, we assumed a priori that 0 and 300 ms after the R-wave would correspond to minimal and maximal baroreceptor activation; even though this assumption appears well-supported by the available literature, we did not confirm its validity, and it is likely that unaccounted inter-individual differences were present to some extent. Additionally, in this study an analysis on the QT intervals was not performed, calling for further investigation in combination with more comprehensive autonomic response monitoring. Fourthly, due to time limitations we did not perform a control examination with non-nociceptive stimuli. As a consequence, even though the observed N2/P2 complex is specific to nociceptive stimuli, we cannot exclude that analogous effects could be present on the responses elicited by weaker, non-nociceptive stimuli.

In the present study, pain delivery during baroreceptor activity was associated with attenuated P2 amplitude only for stimuli preceded by the warning cue. This partially agrees with previous findings indicating that the pain-evoked potentials, namely the N2, P2 or N2-P2 peak-to-peak amplitudes, are reduced during baroreceptor discharge [4,11,26,54]. In our case, this effect was only observed for cued stimuli. We speculate that this selective effect is related to the different subjective experiences of pain for cued and un-cued stimuli, given that the P2 is known to correlate with subjective ratings of pain intensity; however, we cannot explore this hypothesis, because we did not vary pain intensity or measure subjective ratings of individual pain stimuli [4,11,12,22,29,45,54].

**Fig. 4:**
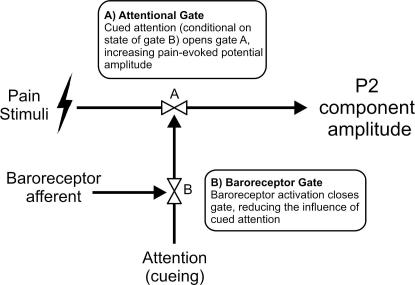
Conceptual model of the interaction between attentional and baroreceptor-related effects on the amplitude of the nociceptive P2 ERP component. There are two “gating” or “modulation” mechanisms. One (A) represents the effect that attentional focus has on the P2, conditional to the state of another mechanism (B), which represents baroreceptor activation. When baroreceptors are quiet, “gate” B is open and the attentional effect can reach A, and thereby modulate the P2 amplitude. However, when the baroreceptors are active, “gate” B blocks the effect of attention, and no cueing-related effect is observed on the P2. Baroreceptor activity does not directly influence the P2 amplitude, it only has an opportunity to do so by gating the attentional signal through B.

Our results are consistent with a conceptual model based on two “gating” or “modulation” processes, wherein expectation of pain acts as a context depending on which baroreceptor activity may or may not attenuate central neural responses to pain (Fig. 4). These results suggest that cognitive attentional influences on pain may be gated by phasic afferent signals from the heart and vasculature. This extends the existing literature in which baroreceptor gating of sensory processes is restricted to brainstem nuclei, without interactions with cortico-limbic processing. The absence of a cardiac timing effect when attention was directed to visual stimuli suggests that cognitive processes contribute to the influence of baroreceptor activation on nociception. One interpretation is that the “analgesic” effects of baroreceptor activation obligatorily require salient or attentionally-focused pain. This is consistent with Donadio’s [20] findings, in that the first stimulus in each train of nociceptive stimuli may have been more attentionally salient than the predictable and regular subsequent ones, especially given that a long inter-stimulus interval (i.e., effectively 60 s) was used. In our study, the period of expectancy and the overall inter-stimulus intervals were kept brief, nevertheless, the explicit cues which always predicted painful stimulation likely increased salience and attention for the cued stimuli. Taken together, these two studies therefore suggest that attention may be more relevant to baroreceptor mediated-attenuation of nociception than a homeostatically-imposed refractory boundary at the level of sympathetic outflow. This interpretation must, however, remain speculative, as our results only pertain to pain-evoked potentials and we did not specifically assess the activity of sympathetic nerves.

Our experimental paradigm did not evoke significant peripheral physiological (i.e. heart rate and blood pressure) change, either of cue-related expectancy or nociceptive stimulation. This might be related to the short inter-stimulus time (i.e., approximately 7 s) or due to the fact that, in contrast with the previous work, here participants were engaged in recognizing visual stimuli rather than passively waiting for nociceptive stimulation [39]. Importantly, the absence of peripheral physiological effects ensured that the observed interaction was genuinely between neural aspects of the expectancy state and cardiac timing, rather than directly explainable in terms of expectancy-related systematic physiological changes.

Our cued pain paradigm also has similarities to paradigms seeking to differentiate “fear” from “anxiety” influences on nociception [57,61]. These studies suggest that probable but unpredictable pain (un-cued pain in our experiment) activates “anxiety” circuitry encouraging hypervigilant evaluation of external and somatic environments [30], whereas directly cued imminent pain activates “fear” circuitry inducing fight/fight responses. Activating “anxiety circuitry” increases pain sensitivity and hyperalgesia [57,67], whereas activating “fear circuitry” decreases pain sensitivity via cortico-limbic [36,40,52], midbrain [46,66] and spinal [2,47] analgesic mechanisms. Our finding however, that P2 amplitude is increased to directly cued pain is inconsistent with this literature, and appears instead to be more directly related to attentional phenomena [73].

A fundamental question pertains to the functional anatomical substrates of the observed interaction. Source localization studies and intracranial recordings have shown that pain-related evoked potentials collectively originate from the pre- and post-central gyri, the anterior cingulate cortex and the insula [6,32,33,65,69–71]. While the pre- and post-central gyri receive nociceptive stimuli only through the thalamus (lateral pathway), nociceptive stimuli propagate to the anterior cingulate cortex, insula [43] and amygdala also through the rostro-ventral medulla and the periacqueductal gray matter (medial pathway;[74]). At its simplest, the baroreflex is a vagal and brainstem-mediated reflex ensuring beat-to-beat alterations in blood pressure remain within homoeostatically defined boundaries [44]. However, baroreceptor afferent information is also transmitted to regions including the insula, which is also implicated in representating cardiovascular afferent information [37–39,77]. We have previously demonstrated that baroreceptor influences on cardiovascular pain reflexes are associated with activity within the periaqueductal gray matter (PAG), amygdala and insula [39]. Functional imaging also reveals attention-related activity within regions including the amygdala, insula and prefrontal cortex [9] may directly influence nociceptive pain processing [56,73]. Further, the potential for lesions within PAG [10], amygdala [40] and prefrontal cortex [52] to influence antinociception implicates these regions as candidates mediating the interaction between attention and cardiac afferents on nociception.

One can broadly hypothesize two complementary but not mutually-exclusive mechanisms: (1) cortical and paralimbic (cognitive-emotional) interactions with baroreceptor afferents in the thalamus, brainstem or PAG and (2) an interaction within cortical and paralimbic regions themselves, including the amygdala, insula and cingulate cortex. Future brain imaging studies are necessary to extend our findings and specifically delineate the neural systems which mediate the observed interaction.

In summary, our study provides what is, to our knowledge, the first evidence that baroreceptor activity interacts with expectancy induced by a visual warning cue. Namely, expectancy determines the influence of baroreceptor activity on central signatures of nociception. In parallel, baroreceptor discharge appears to interfere with the effect of expectancy on nociceptive processing. The effects of expectancy and visceral context on nociception therefore appear closely integrated, suggesting that baroreceptor function may interact with other aspects of cognitive-emotional function. Our findings also contribute to understandings of anti-nociceptive mechanisms, with relevance to the combined influence of mental and physiological contributions to clinical pain.

## Conflict of Interest

We have no financial or other relationships which might represent a conflict of interest.

## Figures and Tables

**Table 1:** Effect of expectancy and cardiac timing on physiological measures. “Silent” and “Active” refer to baroreceptor status. Values are given as mean ± SD. In order to account for multiple comparisons, Bonferroni’s correction indicates *α* = 0.004.

	Stim Beat	1	2	3
*Cardiac cycles post-stimulus*				
*Systolic pressure/ΔmmHg*				
Cued pain, silent	0.2 ± 0.4	−0.1 ± 0.9	0 ± 1.1	−0.1 ± 1.2
Cued pain, active	−0.3 ± 0.7	−0.7 ± 0.8	−0.3 ± 1.4	−0.3 ± 1.8
Un-cued pain, silent	−0.2 ± 0.6	−0.2 ± 1	−0.4 ± 1.1	−0.5 ± 1.2
Un-cued pain, active	−0.3 ± 0.8	−0.5 ± 0.9	−0.4 ± 0.6	−0.3 ± 1
Expectancy	*P* = 0.3	*P* = 0.9	*P* = 0.6	*P* = 0.7
Timing	*P* = 0.2	*P* = 0.1	*P* = 0.5	*P* = 0.9
Timing × expectancy	*P* = 0.01	*P* = 0.4	*P* = 0.5	*P* = 0.4

*Diastolic pressure/ΔmmHg*				
Cued pain, Silent	−0.1 ± 0.4	−0.2 ± 0.6	−0.2 ± 0.9	0.1 ± 0.9
Cued pain, Active	−0.1 ± 0.4	−0.4 ± 0.8	−0.5 ± 1.0	−0.2 ± 1.1
Un-cued pain, Silent	−0.1 ± 0.4	−0.4 ± 0.5	−0.4 ± 0.7	−0.4 ± 0.6
Un-cued pain, Active	−0.2 ± 0.2	−0.3 ± 0.3	−0.3 ± 0.5	0 ± 0.4
Expectancy	*P* = 0.7	*P* = 0.8	*P* = 0.9	*P* = 0.6
Timing	*P* = 0.6	*P* = 0.9	*P* = 0.8	*P* = 0.9
Timing × Expectancy	*P* = 0.4	*P* = 0.6	*P* = 0.4	*P* = 0.2

*Heart rate/Δbpm*				
Cued pain, Silent	−0.7 ± 1.1	−0.2 ± 0.9	0.2 ± 1.2	0.3 ± 1.1
Cued pain, Active	−0.3 ± 1.0	−0.7 ± 0.9	0 ± 1.2	0.2 ± 1.5
Un-cued pain, Silent	−0.3 ± 0.9	0.2 ± 1.2	0.4 ± 1.2	0.6 ± 1.3
Un-cued pain, Active	−0.4 ± 0.9	−0.5 ± 1	0.1 ± 1.3	0.2 ± 1.6
Expectancy	*P* = 0.7	*P* = 0.4	*P* = 0.5	*P* = 0.7
Timing	*P* = 0.6	*P* = 0.02	*P* = 0.4	*P* = 0.5
Timing × Expectancy	*P* = 0.3	*P* = 0.6	*P* = 0.9	*P* = 0.7

**Table 2:** ERP component amplitudes. Values are given in microvolt (μV), as mean ± SD.

	*Cued stimuli*			*Un-cued stimuli*		
	*C3*	*Cz*	*C4*	*C3*	*Cz*	*C4*
*N2 component*						
Baroreceptor Silent	−5.6 ± 3.8	−7.5 ± 4.7	−4.5 ± 3.5	−5.6 ± 4.3	−7.7 ± 5.1	−4.6 ± 3.7
Baroreceptor Active	−6.0 ± 4.3	−7.9 ± 5.2	−4.5 ± 3.7	−4.3 ± 4.0	−7.5 ± 4.9	−4.3 ± 3.6

*P2 component*						
Baroreceptor Silent	4.0 ± 2.2	4.8 ± 2.7	3.1 ± 2.1	3.4 ± 2.0	4.1 ± 2.5	2.3 ± 2.1
Baroreceptor Active	3.4 ± 1.9	4.0 ± 2.3	2.5 ± 1.9	3.7 ± 2.1	4.3 ± 2.5	2.6 ± 2.2
